# Kinin-B2 Receptor Mediated Neuroprotection after NMDA Excitotoxicity Is Reversed in the Presence of Kinin-B1 Receptor Agonists

**DOI:** 10.1371/journal.pone.0030755

**Published:** 2012-02-10

**Authors:** Antonio H. Martins, Janaina M. Alves, Dinely Perez, Marimeé Carrasco, Wilmarie Torres-Rivera, Vesna A. Eterović, Pedro A. Ferchmin, Henning Ulrich

**Affiliations:** 1 Departamento de Bioquímica, Instituto de Química, Universidade de São Paulo, São Paulo, Brazil; 2 Departmento de Neurologia/Neurocirurgia, Universidade Federal de São Paulo, São Paulo, Brazil; 3 Department of Biochemistry, Universidad Central del Caribe, Bayamón, Pueto Rico, United States of America; Florida International University, United States of America

## Abstract

**Background:**

Kinins, with bradykinin and des-Arg^9^-bradykinin being the most important ones, are pro-inflammatory peptides released after tissue injury including stroke. Although the actions of bradykinin are in general well characterized; it remains controversial whether the effects of bradykinin are beneficial or not. Kinin-B2 receptor activation participates in various physiological processes including hypotension, neurotransmission and neuronal differentiation. The bradykinin metabolite des-Arg^9^-bradykinin as well as Lys-des-Arg^9^-bradykinin activates the kinin-B1 receptor known to be expressed under inflammatory conditions. We have investigated the effects of kinin-B1 and B2 receptor activation on N-methyl-D-aspartate (NMDA)-induced excitotoxicity measured as decreased capacity to produce synaptically evoked population spikes in the CA1 area of rat hippocampal slices.

**Principal Findings:**

Bradykinin at 10 nM and 1 µM concentrations triggered a neuroprotective cascade via kinin-B2 receptor activation which conferred protection against NMDA-induced excitotoxicity. Recovery of population spikes induced by 10 nM bradykinin was completely abolished when the peptide was co-applied with the selective kinin-B2 receptor antagonist HOE-140. Kinin-B2 receptor activation promoted survival of hippocampal neurons via phosphatidylinositol 3-kinase, while MEK/MAPK signaling was not involved in protection against NMDA-evoked excitotoxic effects. However, 100 nM Lys-des-Arg^9^-bradykinin, a potent kinin-B1 receptor agonist, reversed bradykinin-induced population spike recovery. The inhibition of population spikes recovery was reversed by PD98059, showing that MEK/MAPK was involved in the induction of apoptosis mediated by the B1 receptor.

**Conclusions:**

Bradykinin exerted protection against NMDA-induced excitotoxicity which is reversed in the presence of a kinin-B1 receptor agonist. As bradykinin is converted to the kinin-B1 receptor metabolite des-Arg^9^-bradykinin by carboxypeptidases, present in different areas including in brain, our results provide a mechanism for the neuroprotective effect in vitro despite of the deleterious effect observed in vivo.

## Introduction

Stroke is a leading cause of death and disability in industrialized countries. Moreover, surviving individuals are often permanently disabled causing major economic losses [1] and immeasurable human suffering. Neuronal cell death by apoptosis or necrosis follows arterial obstruction. The ischemic core is defined by the area where blood flow is completely interrupted and cell death occurs as a result of lack of glucose and oxygen. Neuronal death in the adjacent penumbral zone results from dramatic increases in extracellular concentration of glutamate and augmented stimulation of the NMDA subtype of glutamate receptor resulting in massive influx of calcium [2], [3]. Attempts to block excitotoxic neuronal damage as consequence of ischemia with NMDA receptor antagonists have failed so far due to unexpected effects, which include blockade of inhibitory neurotransmission (for a review [4]) and the unintended inhibition of the pro-survival effect induced by the NMDA receptor [5].

In addition to mechanical or enzymatic removal of the occlusion underlying ischemia, therapeutic approaches aim at protection against neuronal death in the penumbral zone by activation of anti-apoptotic pathways. Kallikrein, an enzyme which releases bradykinin (BK) and kallidin (Lys-BK) after proteolytic cleavage of kininogens, was already shown to participate in neuroprotective effects *in vitro*. Kallikrein gene transference by an adenovirus carrier through intracerebroventricular injection into a rat model of ischemic stroke reduced deficits in motor function by neuroprotection involving promotion of cell survival and migration as well as inhibition of apoptosis by activation of the anti-apoptotic Bcl-2 through AKT and reduction of oxidative stress [6]. These beneficial effects were not only mediated by prolonged reduction of blood pressure, regulation of angiogenesis and neurogenesis in the heart, but also by regulation of AKT-Glycogen synthase kinase (GSK)-3β and activation of AKT-Bad 14-3-3 signaling pathways [7], [8]. Experimental evidence has suggested the involvement of the kallikrein-kinin system in mechanisms of neuroprotection after stroke. For instance, neuroprotection by kinin-mediated promotion of migration and inhibition of apoptosis in primary culture of glial cells was blocked in the presence of the selective kinin-B2 receptor (B2BKR) antagonist HOE-140 (Icatibant) [6].

In this paper we provide evidence for BK-induced neuroprotection of hippocampal neurons against NMDA-mediated excitotoxicity, determined by electrophysiological measurements of recovery of population spikes (PSs) whose magnitudes are directly proportional to the number of synaptically elicited axon potentials by pyramidal neurons [9]. B2BKR-mediated neuroprotection involved phosphatidylinositol kinase (PI-3K) activation, while inhibition of mitogen-activated protein kinase (MEK/MAPK) signaling did not interfere with the induced neuroprotective effects. However, MEK/MAPK activation was involved in kinin-B1 receptor (B1BKR)-mediated signaling which reverted BK-induced PS recovery.

## Results

### The decrease of population spikes is correlated with apoptotic events

**Figure 1:**
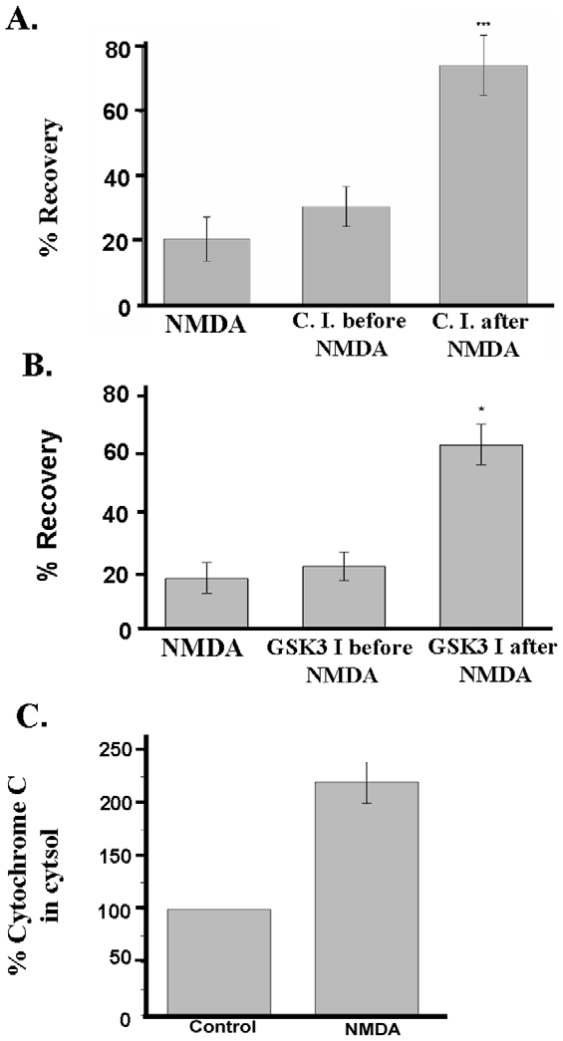
Relation of population spike recovery with apoptosis rates. Initial population spikes (PS) were recorded in *stratum pyramidale* region of hippocampal slices prior to and following a 10 min application of 0.5 mM NMDA. A) Z-LEHD-FMK, a cell-permeant caspase 9 inhibitor (C.I.), was superfused during 1 h prior to NMDA administration or during 1 h after NMDA. Each lane was superfused for 1 h with ACSF, and the initial PS was recorded from seven slices per lane. For the NMDA lane, the perfusion with ACSF continued for 1 h. Then 0.5 mM NMDA was applied for 10 min; the second lane was superfused with 5 µM of the caspase 9 inhibitor for 1 h after NMDA washout; the third lane was superfused with the inhibitor for 1 h prior to exposure to 0.5 mM NMDA for 10 min. After that, all three lanes were superfused with ACSF for 1 h, and at the end of this time, the final PS was recorded. PS recovery rates (peak areas) obtained in the NMDA alone were compared with those obtained in the presence of 0.5 mM NMDA plus 5 µM Z-LEDH-FMK (C.I.) (n = 21, ***, *p*<0.001, as analyzed by Student's t-test). B) The same protocol describe above was used for GSK-3 inhibition (GSK I) by 25 µM SB-216763 (n = 21, * *p*<0.05). C) NMDA-triggered excitotoxicity induces cytochrome c release from mitochondria and is correlated with the decrease of PS areas. Cytochrome c release was measured after 3 hours. The amount of cytochrome c released after NMDA was more than 2 fold greater (220±20%) than detected in control fraction obtained from slices superfused with ACSF (n = 3, p<0.05). (A) to (C): Data are presented as mean values ± standard deviation (S.D.).

Excitotoxicity in acute hippocampal slices was induced by 10 min of exposure to 0.5 mM NMDA and resulted in a reduction of population spikes (PSs) of pyramidal neurons to 25.9±6.9% (mean ± S.E.M.) (Figure 1A; Figure S1A, B) of control recordings from slices which had not been exposed to NMDA. When acute slices had been pretreated with 5 µM Z-LEHD-FMK [Z-Leu-Glu(OMe)-His-Asp(OMe)-FMK.TFA], a caspase 9 inhibitor-II, prior to the NMDA-mediated insult, PSs were not rescued (32.3±6.6% of control values). However, application of Z-LEHD-FMK after NMDA treatment led to a significant improvement in PS recovery (70.2±8.8%, p<0.001) (Figure 1 A). Similarly, population spike recovery occurred when the GSK-3 inhibitor SB-216763 was applied after the NMDA insult (62.8±7.1%), while no protection was observed when the inhibitor was added prior to NMDA application (20.9±4.7% of control measurements, Figure 1B). The release of cytochrome c from mitochondria is one of several signs of apoptosis [10]. Therefore, we measured cytochrome c release in hippocampal slices treated with NMDA, resulting in a decrease of population spikes. NMDA-treated cell preparations released more than twice the amount of cytochrome c more than untreated control cells (Figure 1C).

### Population spikes, decreased in the presence of NMDA, are restored by bradykinin treatment

**Figure 2:**
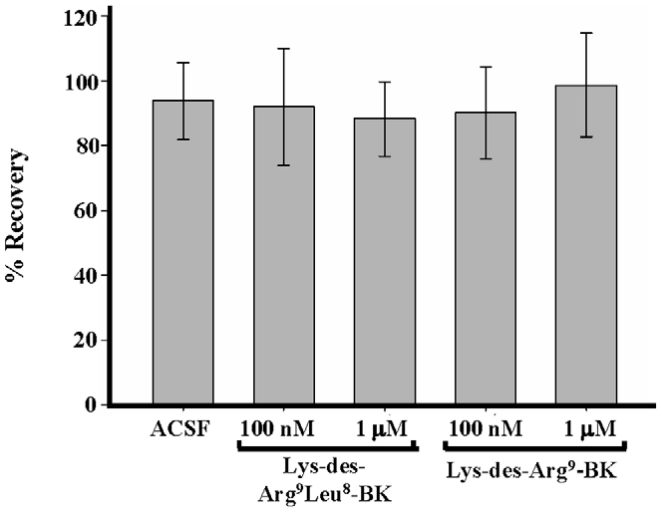
Verification of population spike recovery in the presence of kinin-B1 receptor agonists and antagonists. Synaptically elicited PSs were recorded in the stratum pyramidale region in the absence of NMDA. Control slices were treated only with ACSF and compared with slices treated with 100 nM or 1 µM concentrations of the B1BKR receptor antagonist Lys-des-Arg^9^-Leu^8^-BK or agonist Lys-des-Arg^9^-BK. PSs were measured before and after the application of peptides. Data are presented as mean values ± S.D. (n = 28).

**Figure 3:**
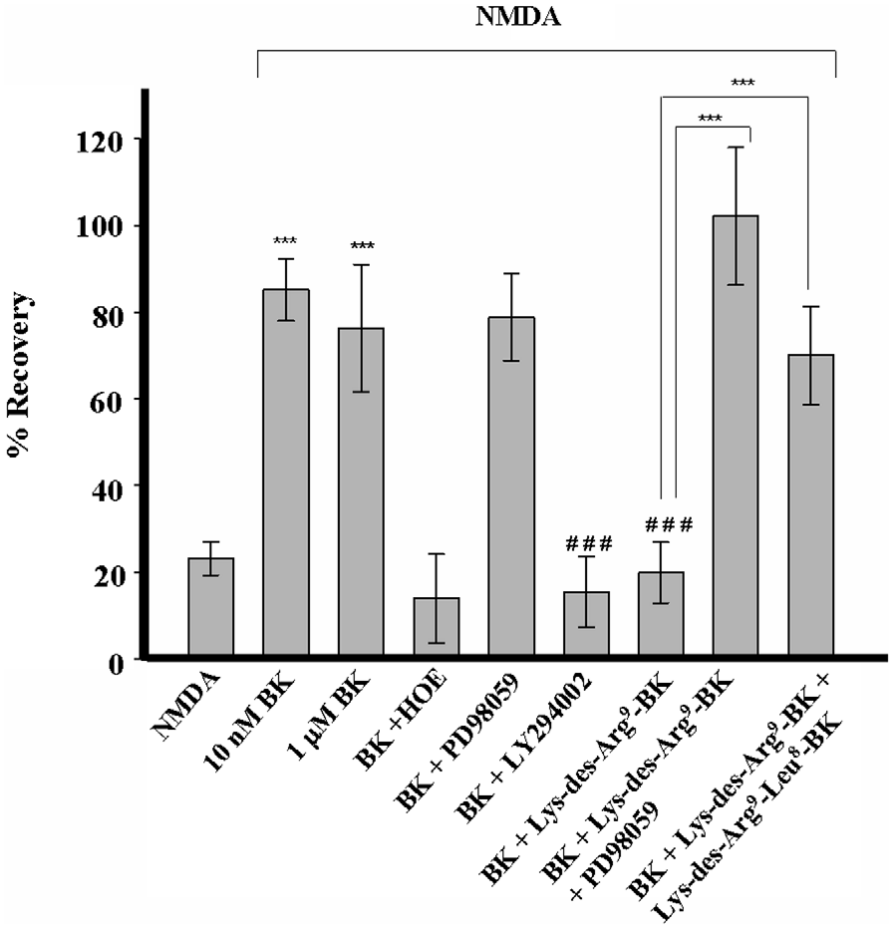
Kinin-B2 receptor mediated protection against NMDA-induced excitotoxicity and its reversion by Lys-des-Arg^9^-bradykinin. Peak areas of synaptically elicited population spikes (PSs) recorded in the *stratum pyramidale* region of hippocampal slices are reported as mean values ± S.E.M. Bradykinin (BK) (10 nM and 1 µM) protected against NMDA (0.5 mM)-mediated cytotoxicity (n = 21, *** p<0.001, compared to control values in the presence of NMDA alone). Neuroprotection induced by 10 nM BK was abolished in the presence of 100 nM HOE-140 (HOE) (n = 21, ### p<0.001, values obtained in the presence of NMDA and BK compared to those collected in the presence of NMDA, BK and HOE-140). The MEK/MAPK inhibitor PD98059 (50 µM) did not interfere with BK-mediated neuroprotection. The PI3-kinase inhibitor LY294002 (10 µM) co-applied with 10 nM BK blocked neuroprotection conferred by BK (n = 21, p<0.05, compared to control values in the presence of BK alone). BK (10 nM)-exerted effects were abolished by 100 nM of the B1BKR agonist Lys-des-Arg^9^-BK (p<0.001, compared to control values in the presence of BK alone). Lys-des-Arg^9^-BK-mediated blockade of neuroprotection was reverted in the presence of PD98059 (50 µM) or the B1BKR antagonist Lys-des-Arg^9^-Leu^8^-bradykinin (1 µM) (p<0.001, compared to control values in the presence of BK and Lys-des-Arg^9^-BK) (n = 21). Statistical analysis was done by one way ANOVA followed by the Dunn's method.

In the absence of NMDA, agonists and antagonists of the kinin-B1 receptor did not affect the magnitude of PS recovery (Figure 2). Application of artificial cerebrospinal fluid (ACSF) for 3 h resulted in 93.6±5.9%, 10 nM of Lys-des-Arg^9^-BK in 90.0±7.0%, 100 nM Lys-des-Arg^9^-BK in 98.6±8.0, 100 nM Lys-des-Arg^9^-Leu^8^-BK in 91.9±9.0% and 1 µM Lys-des-Arg^9^-Leu^8^-BK in 88.2±5.7% of initial PS areas. However, BK at 10 nM and 1 µM protected against NMDA-induced excitotoxicity with recovery rates of 85.3±3.5% and 76.3±7.3%, respectively (Figure 3; Figure S1A–D). As there was no statistical significant difference in the PS recovery at both BK concentrations, 10 nM BK was used for subsequent experiments. BK-evoked protection against NMDA-induced cell death was due to B2BKR activation, since effects mediated by10 nM BK were abolished by co-application of the selective B2BKR receptor agonist HOE-140 (100 nM HOE-140) with 13.9±5.1% of recovery rates measured in control cells (Figure 3; Figure S1A,B,E).

Protection against NMDA-induced cell death may occur by activation of PI3 kinase and MEK/MAPK signaling pathways among other mechanisms. In the presence of 10 µM LY294002, a selective PI3 kinase inhibitor [11], [12], BK-induced recovery of PSs was reduced to values observed in the presence of NMDA alone (15.4±4.0%, p<0.001) (Figure 3; Figure S1A,B,F). The presence of 50 µM PD98059, an inhibitor of MEK/MAPK activation [13], [14], did not interfere with BK-mediated PS recovery indicating that B2BKR receptor evoked neuroprotection did not depend on activation of the MEK/MAPK pathway (Figure 3; Figure S1 A,B,G). Intriguingly, the neuroprotection induced by 10 nM BK was completely inhibited by 100 nM Lys-des-Arg^9^-BK, a B1BKR receptor agonist (19.9±3.5% of control responses, p<0.001). Population spikes were reestablished when 1 µM of the B1BKR receptor antagonist Lys-des-Arg^9^-Leu^8^-BK was applied in combination with BK and Lys-des-Arg^9^-BK (69.9±5.7%, p<0.001). BK-induced neuroprotection was fully restored after application of 50 µM PD98059 together with 10 nM BK and 100 nM Lys-des-Arg^9^-BK (102±7.9% of control responses, p<0.001) indicating that B1BKR action depended on MEK activity.

## Discussion

Glutamate-mediated excitotoxicity involving the loss of calcium homeostasis, oxidative stress and impairment of mitochondrial metabolism (reviewed in [2]) following oxygen and glucose deprivation is the main cause of delayed neuronal loss following the initial cell death in the necrotic core. Glutamate-induced cell death can be reproduced in culture. In cortical neuron cultures, application of 500 µM of the glutamate receptor agonist NMDA induced cell death [15]. The electrophysiological assay used in this work to assess the percentage of functional pyramidal neurons reports well initial phases of ischemic events in hippocampus [3]. Hippocampal slices were already employed in several *in vitro* studies to determine NMDA-induced cell damage and neuroprotective properties of various compounds [16], [17], [18]. In this work, we have shown that PS area is related to apoptosis and cell death, since inhibition of caspase 9 or GSK-3 after the NMDA insult resulted in recovery of PSs. In addition, cytochrome c release in NMDA-treated hippocampal slices was significantly greater when compared with controls (Figure 1). We are the first to show by electrophysiological measurements that NMDA-induced excitotoxic effects on hippocampal neurons can be reverted in the presence of 10 nM or 1 µM BK. BK-induced protection was completely inhibited in the presence of the 100 nM HOE-140, a selective inhibitor of B2BKRs [19].

The effect of BK was thought to be mostly related to regulation of inflammation and blood pressure, but now it is recognized to involve regulation of synaptic functions and neuronal differentiation [20], [21], [22], [23]. B2BKR expression is not limited to endothelial cells in the brain, but is also present along differentiation of rat neural progenitor cells [22], [23]. Developmental processes involve neuroprotective mechanisms resulting in survival of differentiating cells. A recently published study of our group indicated that bradykinin secretion and activation of B2BKR activity were essential for differentiation of P19 embryonal carcinoma cells into neuronal cells expressing functional muscarinic acetylcholine receptors [21]. Neuronal differentiation of this cell line is accompanied by growth factor-mediated inhibition of apoptosis.

In the present paper, we have shown BK-mediated neuroprotection of pyramidal neurons against NMDA-mediated excitotoxicity. Our results indicate that BK-induced recuperation of PSs in hippocampal neurons involved activation of PI3 kinase, which then is responsible for Bad phosphorylation and subsequent anti-apoptotic activity [24]. BK-mediated neuroprotection did not depend on the MEK/MAPK activation cascade. Despite the fact that MEK/MAPK is activated by BK and Lys-des-Arg^9^-BK in transformed airway epithelial cell line cells used as model for allergic airway inflammation [25], this mechanism is not involved in BK-promoted neuroprotection of hippocampus neurons. The observation that inhibition of PI3 kinase by its selective antagonist LY294002, preventing translocation and phosphorylation of downstream proteins, abolished BK-promoted recuperation of PSs is indicative for its involvement in protection against NMDA-mediated excitotoxic effects.

Recent studies suggest the involvement of B2BKRs in neuroprotection in other systems. Danielisova et al. [26], [27] reported that following induction of ischemia, post-conditioning with BK resulted in survival of more than 97% of CA1 neurons. Kallikrein gene transfer reduced apoptosis in a rat model of cerebral ischemia to near-normal levels [6]. Moreover, in agreement with the supposed participation of B2BKRs in neuroprotection, B2BKR receptor knock-out mice subjected to ischemic conditions revealed increased mortality rates and neurological deficits when compared with wild-type animals. Decreased Akt phosphorylation levels correlated with increased apoptosis rates in knock-out animals with arterial occlusion [28].

However, earlier works have connected BK-mediated effects with the induction of postischemic brain damage by evoking increased vascular permeability and subsequent development of brain edema [29], [30]. Further deleterious effects, such as the generation of inflammatory mediators and free radicals [31], appear to be secondary reactions following brain edema formation. Moreover, previous studies suggest the participation of BK in cell death and edema formation following brain ischemia [32], [33], [34]. A rise in expression of B2BKR and an increase of tissue and plasma BK concentration was measured, and inhibition of BK formation decreased edema formation [33]. We hypothesized that a possible mechanism responsible for the harmful effect of BK *in vivo* could be its conversion into des-Arg^9^-BK, an agonist of the B1BKR. In agreement with such hypothesis, 100 nM of the B1BKR agonist Lys-des-Arg^9^-BK completely abolished the neuroprotection provided by bradykinin (Figure 3). Reversion of neuroprotection by Lys-des-Arg^9^-BK was mediated by MEK/MAPK activation, since co-application of 50 µM PD98059 and 10 nM BK restored population spikes. PD98059 is a highly selective inhibitor of MEK/MAPK which activates distinct downstream pathways. The involvement of MEK/MAPK kinases in B1BKR-mediated signal transduction has been described in other systems, such as proliferation induction of breast cancer cells [14], while B2BKRs reveal some pharmacological heterogeneity and the choice of PI-3K and/or MEK/MAPK by the B2BKR depends on the respective cellular context [35].

The deleterious effect exerted by Lys-des-Arg^9^- BK-induced activation of B1BKRs was also blocked in the presence of 1 µM of the selective B1BKR antagonist Lys-des-Arg^9^-Leu^8^-BK. In agreement, a recent study by Austinat et al. [36] provided evidence that blockade of B1BKRs but not of B2BKRs protected against formation of brain edema following ischemic stroke. It is worthwhile to mention that Lys-des-Arg^9^-BK is an adequate compound for studying effects of BK-metabolites on neuroprotection in rats, since this B1BKR agonist and des-Arg^9^- BK possess equal pharmacological profiles in this organism [37]. It is worthwhile mentioning that the cellular origins of kinins and whether B1BKRs and B2BKRs are expressed by the same population of hippocampal neurons needs further investigation. Although kinin receptors are expressed by a variety of neuronal cell lines *in vitro*, *in vivo* neuroprotection may also involve the activation of signaling cascades in astro- and microglial cells such as already suggested by previous work [6], [38].

In addition to providing a molecular mechanism for neuroprotection against excitotoxic actions of NMDA, our work strengthens the statement that B2BKR receptor activity exerts beneficial effects in the central nervous system by promoting survival of neurons, while noxious effects rise from B1BKR activation. Based on its prompt neuroprotective action, a more stable analogue of BK which is not metabolically converted into a B1BKR agonist may turn into a potent therapeutic tool for the treatment of post-ischemic brain damage.

## Materials and Methods

Standard reagents were obtained from Sigma-Aldrich (St. Louis, MO).

### Ethics statement

For protection assays against NMDA-provoked excitotoxicity, hippocampal slices were prepared from male Sprague-Dawley rats (120–200 g) from our colony, which were bred and used following NIH guidelines. Procedures were reviewed and approved by the Institutional Animal Care and Use Committee of Universidad Central del Caribe (Protocol #10-VI-00).

### Cytochrome c release

Cytochrome c release was measured in hippocampal slices using QIA87 Cytochrome C Release Apoptosis Assay Kit (QIAGEN, Valencia, CA) as described by the manufacturer.

### Slice preparation and electrophysiological recordings

Brains from animals sacrificed by decapitation were removed and the hippocampi dissected on ice and irrigated with ice-cold standard artificial cerebrospinal fluid (ACSF) saturated with 95% O_2_, 5% CO_2_ that contained (in mM): 125 NaCl, 3.3 KCl, 1.25, NaH_2_PO_4_, 2 MgSO_4_, 2 CaCl_2_, 25 NaHCO_3_, and 10 glucose. Transversal 400 µm-thick hippocampal slices were cut with a manual slicer and immediately transferred to the incubation chamber and distributed among three lanes with independent perfusion lines. Slices were maintained on a nylon mesh at a temperature of 34±1°C in the interface between ACSF saturated with 95% O_2_, 5% CO_2_ using the same humidified gaseous phase. The temperature was strictly kept constant to avoid variability of results due to temperature-dependent excitotoxicity of glutamate. Slices were allowed to recover from dissection for one hour before starting the electrophysiological recordings. A bipolar electrode placed in the stratum radiatum was used to stimulate the Shaffer collateral incoming fibers with a constant current for 0.2 ms. Synaptically elicited population spikes (PSs) were recorded in stratum pyramidale with a glass electrode filled with 2 M NaCl, having an impedance of 1–5 MΩ. The testing procedure for neurotoxicity was performed according to Schurr et al. [39]. About 30 slices from the hippocampi of two rats were distributed equally among three lanes of the incubation chamber. A maximum of seven slices were analyzed per lane for each replication and 14 to 28 slices were tested per each experimental condition.

One hour after dissection, each slice was stimulated with a pulse twice the strength required to elicit a threshold PS. The initial response was recorded as PS area (ms×mV) and compared to the final response elicited by identical stimulus strength recorded from the same slice after the experimental treatment was finished. The excitotoxic stimulus was delivered by incubation for 10 min with 0.5 mM NMDA in the presence of 95% O_2_, 5% CO_2_ and 10 mM glucose. This concentration of NMDA is known to cause delayed cell death in neuronal cultures [15]. The NMDA concentration and length of exposure to NMDA were set to recover an average of 20% of the PS area after NMDA treatment [16]. Besides NMDA, all other drugs were applied for one hour. The percentage of the initial response remaining at the end of the experiment was used as a measure of electrophysiological recovery of the slice. Slices were incubated with BK at 10 nM and 1 µM concentrations, 10 nM and 100 nM of the B1BKR agonist Lys-des-Arg^9^-bradykinin, 100 nM and 1 µM of the B1BKR antagonist Lys-des-Arg^9^-Leu^8^-bradykinin, 10 µM of the PI3 kinase inhibitor LY294002, 50 µM of the MEK/MAPK inhibitor PD98059 following 0.5 mM NMDA application or with 5 µM of the caspase 9 inhibitor-II Z-LEHD-FMK or 25 µM of the GSK-3 inhibitor SB-216763 prior or following the addition of NMDA. Before the determination of final PSs, the slices were washed with ACSF for one hour to eliminate lingering drugs and any short-living drug effects.

### Data collection and analysis

The areas of PSs were acquired using a GRASS®P5 series AC – pre-amplifier and analyzed with the Labman program (gift from Dr. T.J. Teyler, WWAMI Medical Education Program, University of Idaho, Moscow, ID). The data were statistically analyzed with SigmaStat v11 (SPSS, Chicago, IL). One-way analysis of variance (ANOVA) followed by the Student-Newman-Keuls test was used whenever the data were distributed normally. In some experiments, a large proportion of slices treated with NMDA had zero recovery and the data failed the normality test. In these cases, the less powerful nonparametric Kruskal-Wallis one-way ANOVA on ranks was used followed by the Dunn's test.

## Supporting Information

Figure S1 **Representative traces of population spikes obtained following application of NMDA or NMDA in the presence of BK and/or inhibitors of BK-induced signaling pathways.** Synaptically elicited population spikes (PSs) were recorded in the stratum pyramidale region of hippocampal slices prior and following application of NMDA or NMDA in the presence of BK and/or inhibitors of BK-induced signaling pathways as detailed in the Methods' section. (**A.**) A control slice treated only with ACSF, followed by application of 0.5 mM NMDA alone (**B**), or with 0.5 mM NMDA in the presence of 1 µM BK (**C**) or 10 nM BK (**D**), 10 nM BK and 100 nM HOE-140 (B2BKR antagonist) (**E**), 10 nM BK and 10 µM LY294002 (PI3-kinase inhibitor) (**F**), or with 10 nM BK and 50 µM PD98059 (MEK/MAPK inhibitor) (**G**).
(TIF)
