# Supplementary figures and images for: Kinin-B2 Receptor Mediated Neuroprotection after NMDA Excitotoxicity Is Reversed in the Presence of Kinin-B1 Receptor Agonists
Source: PLoS One. 2012 Feb 10;7(2):e30755. doi: 10.1371/journal.pone.0030755 (PMC3277507; doi:10.1371/journal.pone.0030755)

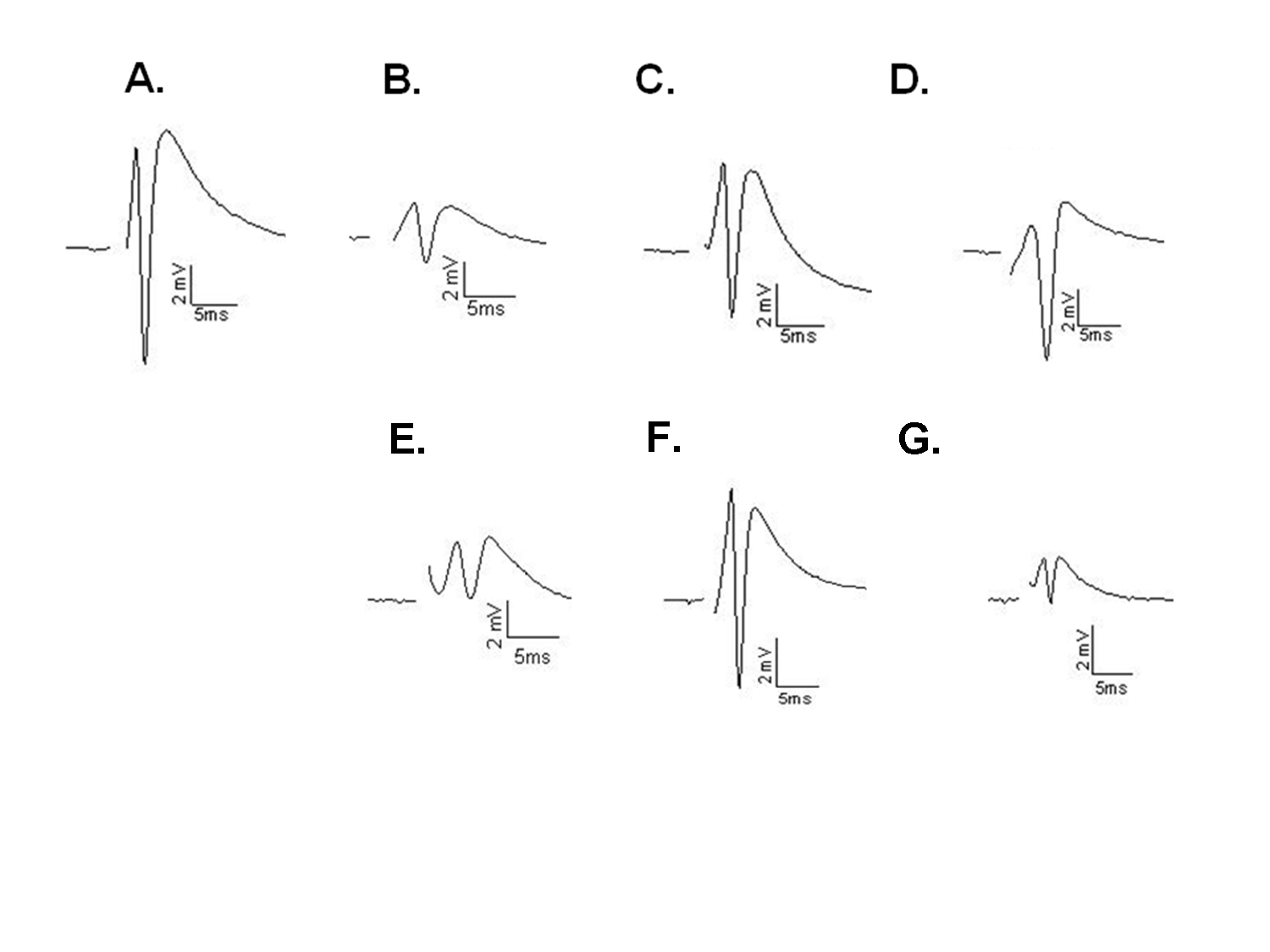

Supplement: Figure S1 — Representative traces of population spikes obtained following application of NMDA or NMDA in the presence of BK and/or inhibitors of BK-induced signaling pathways. Synaptically elicited population spikes (PSs) were recorded in the stratum pyramidale region of hippocampal slices prior and following application of NMDA or NMDA in the presence of BK and/or inhibitors of BK-induced signaling pathways as detailed in the Methods' section. (A.) A control slice treated only with ACSF, followed by application of 0.5 mM NMDA alone (B), or with 0.5 mM NMDA in the presence of 1 µM BK (C) or 10 nM BK (D), 10 nM BK and 100 nM HOE-140 (B2BKR antagonist) (E), 10 nM BK and 10 µM LY294002 (PI3-kinase inhibitor) (F), or with 10 nM BK and 50 µM PD98059 (MEK/MAPK inhibitor) (G). (TIF) [file pone.0030755.s001.tif]
